# Sleep quality impairments in schizophrenia and bipolar affective disorder patients continue during periods of remission: a case-controlled study

**DOI:** 10.5935/1984-0063.20210036

**Published:** 2022

**Authors:** Yunus Hacimusalar, Ozgul Karaaslan, Emre Misir, Ozge Ceren Amuk, Goknur Hacimusalar

**Affiliations:** 1 University of Health Sciences, Kayseri City Hospital, Department of Psychiatry - Kayseri - 38080 - Turkey.; 2 Private System Hospital, Department of Psychiatry, - Kayseri - 38080 - Turkey.; 3 Baskent University, Department of Psychiatry, - Ankara - 06800 - Turkey.; 4 Koç University School of Medicine, Department of Psychiatry - Istanbul - 34450 - Turkey.

**Keywords:** Bipolar disorder, Schizophrenia, Sleep, Sleep Disorders, Circadian Rhythm

## Abstract

**Objective:**

Bipolar disorder (BD) and schizophrenia are chronic psychiatric disorders in which sleep disorders are commonly seen. In mental disorders, residual symptoms may persist even if symptoms are greatly reduced overall. The aim of this study was to compare the sleep quality of schizophrenia and BD patients in remission with that of healthy controls.

**Methods:**

Forty-three patients with schizophrenia, 46 BD patients in remission for at least 3 months, and 51 healthy controls were included the study. The Beck Anxiety Inventory (BAI), Beck Depression Inventory (BDI), Young Mania Rating Scale (YMRS) and Pittsburgh Sleep Quality Index (PSQI) were administered to all participants and the Positive and Negative Syndrome Scale (PANSS) was administered to patients with schizophrenia.

**Results:**

Poor sleep quality was more frequent in the patient groups than the control group (*p*=0.009). PSQI score was positively correlated with duration of disease (*r*=0.236; *p*=0.026), number of cigarettes smoked per day (*r*=0.430; *p*<0.001), body mass index (*r*=0.189; *p*=0.025), and negatively correlated with duration of remission (*r*=-0.224; *p*=0.0359).

**Conclusion:**

Schizophrenia and BD patients in remission had worse sleep quality than a control group. Sleep quality was worst in the patients with schizophrenia. The severity of sleep disorder symptoms was positively associated with disease duration and negatively associated with duration of remission. Schizophrenia and BD patients should be carefully evaluated for symptoms of sleep disorders even when they are in clinical remission and should be offered additional treatment for sleep disorder symptoms when necessary.

## INTRODUCTION

Psychiatric diseases constitute the most extensive diagnostic category among patients with sleep disturbances. Sleep impairments are part of the diagnostic criteria for many psychiatric diseases and commonly co-occur. Sleep impairment emerges before the psychiatric disease becomes full-blown and follows a persistent course even after the disease goes into remission. At the same time, sleep impairment is a deteriorating cause in the recurrence of episodes of chronic psychiatric diseases, particularly bipolar disorder and schizophrenia.

Bipolar disorder (BD) is a disorder interspersed with periods of remission, involving recurrent manic, depressive, and mixed episodes. Sleep disturbances are common during episodes and are among the main symptoms of the disease. It has also shown that sleep impairment plays a significant role in disease susceptibility. A recent community-based study revealed shorter sleep time and increased daytime sleepiness in individuals with hypomanic symptoms^1^. Studies suggest that sleep impairments also continue in the remission phase^2-4^. Meta-analyses evaluated studies with sleep diaries and sleep scales revealed sleep delay, fragmentation, and more inadequate sleep efficiency in patients diagnosed with bipolar disorder than healthy controls^5,6^. These impairments have been shown to continue in the remission period in these studies. Moreover, disturbances in sleep quality cause cognitive symptoms and worsening of the course of the disease^7,8^. Also, the presence of sleep disturbance may trigger BD episodes^9^. At the same time, mood stabilizers, antipsychotics, and antidepressants used in the treatment of the disease will also change sleep quality^10^. However, in a study related to STEP-BD (systematic treatment enhancement program for bipolar disorder), no relationship was found between the use of lithium, valproate, lamotrigine, or atypical/typical antipsychotics and sleep disorders^11^.

Schizophrenia is a chronic disorder characterized by acute psychotic episodes and periods of remission. Sleep disorders are common in schizophrenia, and there is evidence that sleep disorders are related to the severity of symptoms^12,13^. Sleep disorder may appear as an initial symptom, i.e., before developing significant psychotic symptoms, in the initial stages of the disease. It is also common during acute psychotic episodes and significantly affects the disease process^12,13^. However, sleep disorders are not included in the diagnostic criteria for schizophrenia^14^. Sleep disorders may persist during remission^15^, but a little known regarding sleep disorders in patients who are in remission. The relationship between schizophrenia and sleep is not fully understood, and the results of the studies are inconsistent, perhaps due to non-homogeneity of samples, confounding factors, and methodological differences. Patients’ medication may also affect the results of sleep studies^12,16^.

Sleep quality can be affected by many factors other than psychiatric diseases such as stress, medical diseases, nicotine use, age, and body mass index (BMI)^17^. In addition, factors that disrupt sleep, such as medical diseases, obesity, and smoking, are known to increase chronic psychiatric diseases, especially schizophrenia and bipolar disorder^18,19^. It is also known that obesity and smoking are risk factors in terms of obstructive sleep apnea syndrome and insomnia^12^. Therefore, these factors should be taken into account when evaluating sleep disorders in schizophrenia and bipolar disorder.

It is recognized that impairments of sleep quality are common during episodes of bipolar disorder and schizophrenia. However, there is a gap in the literature on sleep quality during periods of remission. The presence of sleep disturbances in the remission phase of both BD and schizophrenia affects the course of these diseases, the risk of recurrence of acute episodes, clinical improvement, quality of life, and cognitive functions^7,11,12,20^.

Sleep studies are often performed with self-report questionnaires, polysomnography (PSG), and actigraphy. In this study, we compared the sleep quality of BD and patients with schizophrenia in remission with that of a healthy control group using a self-report questionnaire. Our first hypothesis is that in the remission period of bipolar disorder and schizophrenia, sleep quality is worse than healthy controls. The second hypothesis is that in bipolar disorder and schizophrenia, sleep quality is negatively correlated with disease duration and positively correlated with duration of remission. In addition, we do not expect to find a difference in sleep disturbance between bipolar disorder and schizophrenia when illness duration and duration of remission are controlled. Our third hypothesis is that sleep quality in remission is negatively affected by residual depressive, manic, and psychotic symptoms. Our fourth hypothesis is that there is a relationship between sleep quality and smoking, body mass index, and antipsychotic use.

## MATERIAL AND METHODS

**Figure 1:**
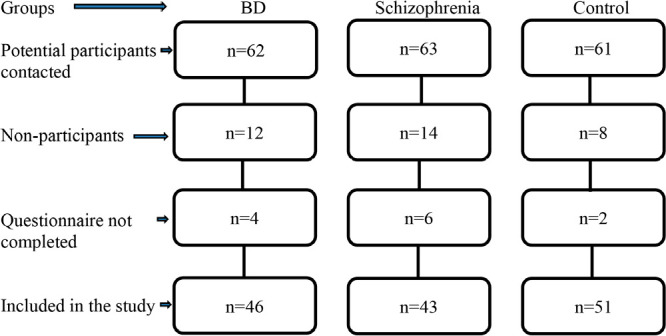
Flow chart of participants. BD: bipolar disorder.

A case-controlled study was performed. Diagnoses of BD and schizophrenia were made by two different specialists (YH, OK) based on DSM-5 criteria^1^. Patients were recruited consecutively at the outpatient psychiatric clinic of the University Faculty of Medicine Hospital and followed up by the same clinic. To be eligible for the study, patients had to meet the following criteria: aged 18-65 years; without comorbid psychiatric disorder; in remission for at least 3 months; capable of completing the tests (self-reported questionnaires). Sleep quality was evaluated with the Pittsburgh Sleep Quality Index (PSQI). All participants also completed the Beck Anxiety Inventory (BAI), Beck Depression Inventory (BDI), and Young Mania Rating Scale (YMRS). Patients with schizophrenia also completed the Positive and Negative Syndrome Scale (PANSS). BD patients were considered to be in remission if they met the following criteria: BDI≤9, BAI≤7, and YMRS≤5. Patients with schizophrenia were considered to be in remission if they met these criteria and scored ≤2 on all PANSS items. The control group consisted of health personnel working in the same hospital and their relatives, and were free from physical and psychiatric disease and sleep disorders. Patients were excluded if benzodiazepine users, pregnant, had chronic physical or psychiatric disorders that may cause sleep disorders, or had alcohol or substance use disorder. After the evaluation of previous study results, a power analysis was performed. The alpha and beta errors were stated, respectively, as 0.05 and 0.20. The minimum number of patients needed to obtain 80% power was calculated as 41 for each group. A total of 140 subjects (43 schizophrenia, 46 BD, and 51 healthy controls) were included in the study (Figure 1).

Individuals who agreed to participate in the study were given information about the objectives and procedures and provided written, informed consent. The study was approved by the ethics committee of the University (protocol number 2017-KAEK-189_2019.02.28_11) and performed following the ethical principles of the declaration of Helsinki for medical research involving human subjects.

### Assessment tools

#### Demographic variables and medical background

Data on age, gender, educational background, duration of disease, duration of current remission period, medication, smoking, height, and weight were collected using a form developed specifically for this study.

#### Pittsburgh Sleep Quality Index (PSQI)

The PSQI was developed by Buysse et al. (1989)^21^, and the validity and reliability of the Turkish version were assessed by Ağargün et al. (1996)^22^. The PSQI is a 24-item scale that assesses sleep quality and sleep disturbance over the past month. It consists of 19 self-report items and 5 items to be completed by the partner of the primary respondent. The PSQI is organized into seven subscales: subjective sleep quality, sleep latency, sleep duration, sleep efficiency, sleep disturbance, use of sleep medication, and daytime dysfunction. Each subscale is rated from 0 to 3 points. The score for the seven subscales is summed to give a total score (0-21 points). A high PSQI total score indicates poor sleep quality and severe sleep disturbance. A total PSQI score of over 5 points indicates clinically poor sleep quality.

#### Beck Depression Inventory (BDI)

This was developed by Beck et al. (1961)^23^ to evaluate the physical, emotional, cognitive, and motivational symptoms seen in depression. The BDI consists of 21 self-report items to which responses are given using a 0-3 scale. Total scores range from 0-63; scores of 0-9 indicate minimal symptoms. The validity and reliability of the Turkish version were assessed by Hisli et al. (1988)^24^.

### Beck Anxiety Inventory (BAI)

The BAI was developed by Beck et al. (1988)^25^ to assess anxiety symptoms. It consists of 21 self-report items scored using a 0-3 scale. Total scores range from 0-63. Higher scores indicate more severe symptoms of anxiety. The validity and reliability of the Turkish version were assessed by Ulusoy et al. (1998)^26^.

### Young Mania Rating Scale (YMRS)

The Young Mania Rating Scale (YMRS) was developed by Young et al. (1978)^27^, and the reliability and validity of the Turkish version were assessed by Karadağ et al. (2001)^28^. The scale consists of 11 self-report items to which responses are given using a 0-4 scale.

### Positive and Negative Syndrome Scale (PANSS)

The PANSS consists of 30 items organized into three subscales (positive symptoms scale, negative symptoms scale, and general psychopathology scale)^29^. It is a semi-structured scale that is intended to evaluate the symptoms and functionality of patients with schizophrenia. The interviewer administered the scale scores for each item using a 1-7 scale, according to the severity of symptoms. A Turkish version of the PANSS was developed by Kostakoğlu et al. (1990)^30^.

### Statistical analysis

Statistical analysis was performed using SPSS 22.0 (Statistical Package for Social Sciences, IBM Inc., Chicago, IL, U.S.). Descriptive statistics were calculated, and the Kolmogorov-Smirnov test was used to assess the normality of variable distributions. The chi-squared test was used for group comparisons of categorical variables. Group differences in normally distributed variables were assessed through ANOVA and post hoc Turkey tests. The Kruskal-Wallis H test was used to compare non-parametric variables, and omnibus effects were explored using the Mann-Whitney U test to determine which pairs of groups differed, and *p* significance was determined by dividing the significance value with the number of groups. Bonferroni correction for multiple comparisons was used (0.05/3=0.017). Associations between variables were assessed with Pearson’s correlation test (normally distributed variables) or Spearman’s correlation test (non-parametric variables). Correlated variables were used in a multiple linear regression model, and diagnostic tests of this model were carried out. The level of significance was set at *p*<0.05 for all tests.

## RESULTS

The mean ages of the groups were similar (*p*=0.124). The mean BMIs of the patient groups were higher than the BMI of the control group (*p*<0.001). Twenty-eight (65.1%) of patients with schizophrenia, 16 (34.8%) of BD patients and 20 (39.2%) controls were male. The proportion of men was higher in the patients with schizophrenia than in the BD group or control group (*p*=0.008). Smoking was more frequent in the patient groups than in the control group (*p*=0.002). The mean number of cigarettes smoked per day was higher among smokers in the schizophrenia group than those in the BD and control groups (*p*<0.001) (Table 1). The median total PSQI scores were similar in the two patient groups and higher in the schizophrenia group than in the control group (*p*=0.004). Sleep quality was poor in 65.1% of patients with schizophrenia, 58.7% of BD patients, and 35.3% of the control group. The prevalence of poor sleep quality was higher in the patient groups than in the control group (*p*=0.009) (Table 2). When a post-hoc power analysis was applied according to the total PSQI scores among the three groups, alpha 0.05, effect size 0.318, and the power of the study was found to be 0.947.

**Table 1:** Comparison of sociodemographic data from the schizophrenic, BD, and control groups.

	Schizophrenia	BD	Control	Comparison	
	(n=43) Mean±SD	(n=46) Mean±SD	(n=51) Mean±SD	F	p^1^
Age in years	42.58±9.75	38.42±11.6	37.64±8.5	2.117	0.124
[min-max]	[23-62]	[24-63]	[18-65]		
BMI	31.34±5.36^a^	28.37±4.63^a^	27.44±4.65	14.497	<0.001
	**n (%)**	**n (%)**	**n (%)**	**x** *^2^*	**p** *^2^*
Female	15 (34.9)	30 (65.2)	31 (60.8)		
Male	28 (65.1)**^b^**	16 (34.8)	20 (39.2)	9.606	0.008
Smokers	26 (60.5)**^a^**	26 (56.5)**^a^**	14 (27.5)	12.623	0.002
	**Median** **(min-max)**	**Median** **(min-max)**	**Median** **(min-max)**	**x** *^3^*	**p** *^3^*
Cigarettes per day	32.5 (1-49)**^b^**	20.0 (3-35)	13.5 (2-30)	16.619	<0.001**
				**Z**	**p^4^**
Duration of disease (months)	230 (36-390)**^c^**	120 (12-360)		-3.287	0.001**
Duration of remission (months)	36 (3-180)	10.5 (396)		-3.637	<0.001**

**Notes:** *p^1^*: ANOVA; *p^2^*: Chi-square; *p^3^*: Kruskal-Wallis; *p*^4^: Mann-Whitney U; BD: Bipolar disorder; n: Number of participants; BMI: Body mass index; SD: Standard deviation;

* *p*<0.05;

** *p*<0.001;^**a**^Higher than the control group;^**b**^Higher than bipolar affective disorder and control groups;^**c**^Higher than bipolar affective disorder group.

**Table 2:** Comparison of sleep quality of schizophrenic, BD, and control groups.

	Schizophrenia	BD	Control	Comparison	
	**(n=43)**	**(n=46)**	**(n=51)**	**F**	**p^1^**
Poor sleep quality	28 (%65.1)^a^	27 (%58.7)^a^	18 (%35.3)	9.494	0.009**
	**Median** **(min-max)**	**Median** **(min-max)**	**Median** **(min-max)**	**x^2^**	**p^2^**
Total PSQI score	10 (0-16)^a^	8.5 (0-16)	6.0 (2-17)	9.126	0.010*
**PSQI subscales**				**x^2^**	**p^2^**
Subjective sleep quality	1 (0-3)	1 (0-3)	1 (0-3)	2,402	0.301
Sleep latency	2 (0-3)	1 (0-3)	1 (0-3)	5.880	0.053
Sleep duration	0 (0-3)^b^	0 (0-3)^b^	1 (0-3)	27.138	<0.001
Usual sleep activity	1 (0-3)^a^	1 (0-3)^a^	0 (0-3)	14.107	0.001
Sleep disorder	1 (0-2)	1 (0-2)^c^	1 (0-2)	10.782	0.005
[mean rank]	[79.48]	[56.93]	[75.17]		
Sleep drug use	1 (0-3)^a^	0 (0-3)^a^	0 (0-2)	28.433	<0.001
Daytime dysfunction	1 (0-3)	1 (0-3)	1 (0-3)	3.767	0.152

**Notes:** *p*^1^: Chi-square; *p*^2^: Kruskal-Wallis; n: Number of participants; BD: Bipolar disorder;

a Higher than the control group;

b Lower than the control group;

c Lower than schizophrenic and control groups.

When the PSQI subcomponents were compared, there were group differences in sleep duration (*p*<0.001), habitual sleep efficiency (*p*=0.001), sleep disturbance (*p*=0.005), and use of sleep medication (*p*<0.001). Paired comparisons made with the Mann-Whitney U test showed that sleep time scores in the schizophrenia and BD groups were lower than the control group (*Z*=-3.777, *p*<0.001; *Z*=-4.786, *p*<0.001, respectively). Typical sleep efficacy scores were higher in the schizophrenia and BD groups than in the control group (*Z*=-3.777, *p*<0.001; *Z*=-2.591, *p*=0.010, respectively). Sleep disturbance scores were lower in the BD group than in the schizophrenia and control groups (*Z*=-2.855, *p*=0.004; *Z*=-2.717, *p*=0.007, respectively). Scores for the use of sleeping drugs were higher in the schizophrenia and BD groups than in the control group (*Z*=-5.332, *p*<0.001; *Z*=-5.380, *p*<0.001, respectively) (Table 2). Total PSQI score was positively correlated with disease duration (*r*=0.236; *p*=0.026) and negatively correlated with duration of the current remission period (*r*=-0.224; *p*=0.035). PSQI score was also positively correlated with the number of cigarettes smoked per day (*r*=0.430; *p*<0.001) and BMI (*r*=0.189; *p*=0.025) (Table 3). Multiple linear regression was used to examine the variables predicting sleep quality (PSQI score). With disease duration, remission time, and BMI as independent variables, remission time and disease duration accounted for 23.5% of the increase in PSQI in BD patients (F(2,43)=7.898; *p*=0.001). With the duration of disease, remission, BMI, and daily cigarette smoking as independent variables, daily cigarette smoking accounted for 13.7% of the variance in PSQI scores (F(1,24)=4.958; *p*=0.036) (Table 4).

**Table 3:** Correlations between PSQI score and other variables investigated.

	PSQI	
	r_s_	p
Duration of disease (months)	0.236	0.026*
Duration of remission (months)	-0.224	0.035*
Cigarettes per day	0.430	<0.001**
BMI	0.189	0.025*

**Notes:** rs: Spearman’s rank correlation coefficient;

* p<0.05;

** p<0.001; PSQI: Pittsburgh Sleep Quality Index; BMI: Body mass index.

**Table 4:** Predictors of PSQI in patients with BD and schizophrenia: multiple linear regression analysis (forward method).

PSQI in BD	adjusted R^2^ (0.235)	R^2^ change	β	F (df)	p
Duration of remission		0.193	-0.479	10.510 (1,44)	0.002
Duration of disease		0.076	0.278	7.898 (2,43)	0.001
**PSQI in schizophrenia**	**adjusted R^2^**		**β**	**F (df)**	**p**
Cigarettes per day	0.137		0.414	4.958 (1,24)	0.036

**Notes:** PSQI: Pittsburgh Sleep Quality Index; BD: Bipolar disorder.

There were overall group differences in scores on the BAI (F(2,137)=6.153; *p*=0.003), BDI (F(2,136)=69.604; *p*<0.001), and YMRS (*p*=0.005). When the differences between groups evaluated by the post-hoc Turkey test indicated that BAI scores were higher in patients with schizophrenia than controls (*p*=0.002). BAI levels of BD and control groups were similar (*p*=0.183). BDI scores were higher in both patient groups than in the control group (*p*<0.001). YMRS scores were also higher in the patient groups than the control group (schizophrenia: *Z*=-3.057, *p*=0.002; BD: *Z*=-2.433, *p*=0.015) (Table 5). In addition, PSQI total score had a statistically significant correlation with PANSS score (*r*=0.322; *p*=0.035) but not with BDI and BAI total scores in schizophrenia patients. Moreover, correlations between PSQI and BDI or BAI statistically significant in patients diagnosed with bipolar disorder (*p*>0.05).

**Table 5:** Comparison of BAI, BDI, YMRS, and PANSS scores in the schizophrenic, BD, and control groups.

	Schizophrenia (n=43)	BD (n=46)	Control (n=51)	Comparison	
	Mean±SD	Mean±SD	Mean±SD	F	p­^1^
BAI	4.19±0.98^a^	3.83±0.90	3.50±0.99	6.163	0.003
BDI	7.26±1.12^a^	6.91±1.11^a^	4.62±1.31	69.604	<0.001
PANSS	46.44±7.27				
	**Median** **(min-max)**	**Median** **(min-max)**	**Median** **(min-max)**	**x^2^**	**p^2^**
YMRS	3 (1-5)^a^	3 (1-5)^a^	2 (1-4)	10.547	0.005

**Notes:**^1^One way ANOVA;^2^ Kruskal Wallis test;

a Higher than the control group; n: Number of participants; BD: Bipolar Disorder; BAI: Beck Anxiety Inventory; BDI: Beck Depression Inventory; YMRS: ; PANSS: Positive and Negative Syndrome Scale.

Fourteen of the BD patients (30.4%) were on mood stabilizer monotherapy, 30 (64.2%) were on mood stabilizer plus antipsychotic combination, and 2 (4.4%) were on antipsychotics alone. Benzodiazepine class drugs were not used in schizophrenia patients, but only one patient with bipolar disorder used diazepam (5mg per day). Almost all patients were on atypical antipsychotics except for three patients. One patient in the bipolar disorder group and two patients in the schizophrenia group used typical antipsychotics. Due to the deficient number of patients using typical antipsychotics, after excluding these patients for comparison sleep qualities among groups according to drug classes, total PSQI scores were found similar in patients who used antipsychotics in addition to mood stabilizers and those who used mood stabilizers alone (*p*=0.128).

## DISCUSSION

When sleep quality was analyzed as a binary variable, based on PSQI score, schizophrenia and BD patients had a higher rate of poor sleep quality than the control group. According to PSQI scores, schizophrenia patients had higher sleep disturbance levels than the control group. Sleep disturbance levels of BD patients were similar to the control group. Disease duration, smoking, and high BMI were negative predictors of sleep quality, whereas remission duration was a positive predictor. In BD patients, the rate of sleep disorders was similar in patients using antipsychotics in addition to mood stabilizers and those using mood stabilizers alone. These findings suggest that schizophrenia and bipolar disorder patients experience poor sleep quality during remission.

Sleep changes in patients diagnosed with schizophrenia mainly involve insomnia or excessive sleepiness. Insomnia is frequently observed during the acute exacerbation of schizophrenia. The sleep-wake cycle may be reversed, and patients may be awake at night and sleepy during the day. Sleep delay, increased number of awakenings, and wakefulness times, total sleep time, and decreased sleep activity are common symptoms in schizophrenia^31,32^. Studies show significant differences between schizophrenia and healthy controls, except for the percentage of stage 2 sleep and the duration of the first REM period, in other characteristics of sleep^33^. The pathophysiology of sleep disturbance in both BD and schizophrenia has not been fully elucidated^16,34,35^. Dopamine (DA) plays an essential role in the pathophysiology of schizophrenia and BD^36,37^. Dopamine also has effects on sleep. D2 receptor agonists such as bromocriptine, pergolide, and apomorphine increase alertness, whereas D2 blocking agents enhance sleep in animals^16^. Glutamate and gamma-aminobutyric acid (GABA) are also involved in the pathophysiology of schizophrenia^36^. Glutamate and GABA may play a role in the pathophysiology of sleep disorders in schizophrenia, but there is not yet enough evidence to confirm this^13,16,38^. Serotonergic, orexinergic, and cholinergic systems are also involved in the pathophysiology of both schizophrenia and sleep disorders^39^.

There are many neurotransmitter systems involved in both schizophrenia and sleep disorders. Complete remission is rare in patients with schizophrenia, although clinical symptoms can be greatly reduced, and persistent dysregulation of neurotransmitter systems may be the cause of symptoms of sleep disturbance during periods of remission^13^. We found that the duration of disease was negatively related to sleep quality, whereas the duration of remission was positively related. Multiple linear regression analyses indicated that duration of disease and length of remission was more strongly related to sleep quality in BD than in schizophrenia. Studies evaluating the relationship between illness duration and sleep in patients with schizophrenia are limited^33^. In a meta-analysis evaluating polysomnography studies in patients with schizophrenia, it was reported that slow-wave sleep percentage and REM sleep percentage were lower in patients with long disease duration compared to patients with shorter disease duration^33^. However, in our study, an evaluation could not be made regarding the sleeping houses, since polysomnographic examination was not performed.

In BD, acute episodes and periods of remission are more clearly differentiated than in schizophrenia. Residual symptoms and chronic features are less common in BD than in schizophrenia, and functioning is less impaired^40,41^. Due to the clinical course of both diseases, the higher incidence of sleep disorder in schizophrenia than in BD is consistent with the clinical features and course of both diseases. So, in our study, patients with BD had lower levels of sleep disturbance than patients with schizophrenia. Besides that, the PSQI score was not correlated significantly with residual symptoms measured with YMRS in patients with bipolar disorder, although residual symptoms measured with PANSS had a statistically significant positive correlation with PSQI in schizophrenia patients. Thus, relatively poor sleep quality in schizophrenia patients may be explained by the impact of residual symptoms. On the other hand, the causality between sleep problems and residual symptoms cannot be suggested because of the cross-sectional nature of the study. Besides, sleep disturbances are in residual symptomatology^42^. There is evidence that in BD the distribution of sleep stages is different during inter-episode periods^6^. However, the results of sleep disturbance studies in remission are still insufficient. In our sample, poor sleep quality was more frequent in the BD group than in the control group, but there was no difference in PSQI scores. This result may be related to the choice of scale and the small sample size.

The therapeutic properties of antipsychotic drugs in schizophrenia are mainly associated with dopamine receptor blockade, but antipsychotics also have effects on serotonin, cholinergic, α-adrenergic, and histamine receptors^32,43^. There are reports that antipsychotic drugs affect sleep^13^ and improve sleep quality in treated patients with schizophrenia^44^. Antipsychotics can cause daytime somnolence and sedation^43^. Antipsychotic drugs are generally divided into the first generation and the second generation. Although the first and second generations are similar in terms of efficacy, they differ in receptor binding affinities and side effects^31^. First-generation antipsychotic drugs possibly reduce stage 2 sleep latency, increase total sleep time and sleep efficiency, and significantly increase REM latency^43^. However, methodological problems and the limited number of studies are fundamental problems. The effects of second-generation antipsychotics on sleep are quite different from each other. For example, olanzapine and paliperidone decrease sleep latency in patients with schizophrenia, increase total sleep time and sleep efficiency, and augmented slow-wave sleep and rapid-eye-movement (REM) sleep. On the other hand, Quetiapine can cause sleep disruptions due to increased sleep latency and REM sleep latency, decreased slow-wave sleep, and REM sleep. During risperidone treatment, a consistent effect on sleep variables was not obtained. The effects of second generation antipsychotics on sleep are quite different from each other^31,43^. Second-generation antipsychotics seem to improve both sleep maintenance and sleep architecture in many schizophrenic patients^32^. Discontinuation of drug treatments in schizophrenia patients also has effects on sleep^33^.

In our study, we found that patients with schizophrenia and BD patients slept longer than the control group. There are other reports that BD patients sleep longer than healthy controls^4,11^. The PSQI treats short sleep duration as a negative aspect of sleep quality but does not consider long sleep duration to impair sleep quality. This is a limitation of relying solely on the PSQI to assess sleep quality. It has been reported that BD patients have difficulty falling asleep, frequently wake during the night, have difficulty waking up in the morning, and have reduced sleep efficiency between attacks^6^. We found that concerning conventional indicators of sleep efficacy and sleeping pill use, BD patients had a worse sleep than controls. Keskin et al. (2018)^2^ that sleep quality was similar in euthymic BD patients taking mood regulators and those who are taking antipsychotics. We found that patients using mood stabilizers alone showed similar levels of sleep disturbance to those taking mood stabilizers plus antipsychotics.

In a study, sleep quality evaluated with PSQI, euthymic BD patients with lithium has better sleep efficiency and longer sleep duration than those without lithium^45^. The mechanism of action of lithium is still unknown^46^. Lithium can affect sleep through many systems such as chronobiological mechanism, phase-delaying properties, and melatonergic system^46^. Most studies in epilepsy patients have shown no significant effects of valproate use on sleep. In some cases, valproate can cause mild sleep disruption^47^. Sylvia et al. (2012)^11^ found no association between sleep disturbance and use of lithium, valproate, atypical antipsychotics, or lamotrigine. The sedative properties of antipsychotics can impair sleep quality, but Yetkin et al. (2011)^48^ reported that unmedicated patients with schizophrenia also had sleep disturbances. This finding supports the deterioration of sleep quality in patients with schizophrenia independent of drugs.

Obesity is seen more frequently in schizophrenia and bipolar patients than in healthy people due to the side effects of psychotropic medication and the reduction in physical activity associated with these diseases. Studies have shown a negative relationship between BMI and sleep quality in both BD^49^ and schizophrenia^50^, which our findings corroborate.

Schizophrenia and bipolar patients are known to smoke more than the general population^12,51^. Patients self-medicate with nicotine to reduce the side effects of their medication and nicotine has effects on mood^51,52^. Smoking is also stimulating. Keskin et al. (2018)^2^ reported that 48.4% of BD patients smoked. In our study, smoking rates were higher in the patient groups than in the control group. We also found that sleep quality was negatively related to the number of cigarettes smoked. Smoking can cause sleep disorders due to both its stimulant properties and its effects on the respiratory system, including exacerbation of respiratory disease symptoms.

### Limitations

The most important limitations of the study are the cross-sectional design and small sample size. Although the PSQI is a validated sleep quality scale, it is not sufficient to assess sleep quality and sub-components of sleep disorder. However, although self-report questionnaires are not diagnostic tools, they can be used to determine if further assessments are needed. Actigraphy and polysomnography provide more detailed information about sleep, but the inconvenience and expense mean that use is not widespread. The drugs used to treat BD and schizophrenia also affect sleep quality. Evaluations of patients who do not use medication during remission periods would be valuable, but since both BD and schizophrenia are chronic, recurrent diseases, it would be challenging to recruit unmedicated patients. The study also has several strengths. Patients who had been in remission for at least 3 months were included in the study and compared with the control group. BMI, smoking, and use of drugs that might affect sleep quality were evaluated.

## CONCLUSION

BD and schizophrenia are severe mental disorders. Sleep disorders are a significant part of both diseases and may persist during remission periods. There are no guidelines for the management of sleep disorders in patients with schizophrenia. The effective treatment of sleep disorder in these patients would help to reduce relapse and recurrence, so it is crucial to evaluate symptoms of sleep disorder in these patients and carry out formal diagnostic investigations and provide treatment where necessary.

## References

[1] Hensch T, Wozniak D, Spada J, Sander C, Ulke C, Wittekind DA (2019). Vulnerability to bipolar disorder is linked to sleep and sleepiness. Transl Psychiatry.

[2] Keskin N, Tamam L, Ozpoyraz N (2018). Assessment of sleep quality in bipolar euthymic patients. Compr Psychiatry.

[3] Ritter PS, Höfler M, Wittchen HU, Lieb R, Bauer M, Pfennig A (2015). Disturbed sleep as risk factor for the subsequent onset of bipolar disorder - data from a 10-year prospective-longitudinal study among adolescents and young adults. J Psychiatr Res.

[4] Harvey AG, Schmidt DA, Scarnà A, Semler CN, Goodwin GM (2005). Sleep-related functioning in euthymic patients with bipolar disorder, patients with insomnia, and subjects without sleep problems. Am J Psychiatry.

[5] Geoffroy PA, Scott J, Boudebesse C, Lajnef M, Henry C, Leboyer M (2015). Sleep in patients with remitted bipolar disorders: a meta-analysis of actigraphy studies. Acta Psychiatr Scand.

[6] Ng TH, Chung KF, Ho FYY, Yeung WF, Yung KP, Lam TH (2015). Sleep-wake disturbance in interepisode bipolar disorder and high-risk individuals: a systematic review and meta-analysis. Sleep Med Rev.

[7] Saunders EF, Novick DM, Fernandez-Mendoza J, Kamali M, Ryan KA, Langenecker SA (2013). Sleep quality during euthymia in bipolar disorder: the role of clinical features, personality traits, and stressful life events. Int J Bipolar Disord.

[8] Bradley AJ, Anderson KN, Gallagher P, McAllister-Williams RH (2020). The association between sleep and cognitive abnormalities in bipolar disorder. Psychol Med.

[9] Harvey AG, Talbot LS, Gershon A (2009). Sleep disturbance in bipolar disorder across the lifespan. Clin Psychol.

[10] Keskin N, Tamam L (2016). Sleep in bipolar disorder. Curr Approaches Psychiatry.

[11] Sylvia LG, Dupuy JM, Ostacher MJ, Cowperthwait CM, Hay AC, Sachs GS (2012). Sleep disturbance in euthymic bipolar patients. J Psychopharmacol.

[12] Kaskie RE, Graziano B, Ferrarelli F (2017). Schizophrenia and sleep disorders: links, risks, and management challenges. Nat Sci Sleep.

[13] Kamath J, Virdi S, Winokur A (2015). Sleep disturbances in schizophrenia. Psychiatr Clin North Am.

[14] American Psychiatric Association (APA) (2013). Diagnostic and statistical manual of mental disorders.

[15] Haffmans PMJ, Hoencamp E, Knegtering HJ, Van Heycopt Ten Ham BF (1994). Sleep disturbance in schizophrenia. Br J Psychiatry.

[16] Monti JM, Monti D (2005). Sleep disturbance in schizophrenia. Int Rev Psychiatry.

[17] Bixler E (2009). Sleep and society: an epidemiological perspective. Sleep Med.

[18] Khalil R (2013). The metabolic syndrome and schizophrenia: a comorbidity or an association?. J Pharmacol Pharmacother.

[19] Daré LO, Bruand PE, Gérard D, Marin B, Lameyre V, Boumédiène F (2019). Co-morbidities of mental disorders and chronic physical diseases in developing and emerging countries: a meta-analysis. BMC Public Health.

[20] Giglio LMF, Andreazza AC, Andersen M, Ceresér KM, Walz JC, Sterz L (2009). Sleep in bipolar patients. Sleep Breath.

[21] Buysse DJ, Reynolds CF, Monk TH, Berman SR, Kupfer DJ (1989). The Pittsburgh sleep quality index: a new instrument for psychiatric practice and research. Psychiatry Res.

[22] Ağargün MY, Kara H, Anlar Ö (1996). The validity and reliability of the Pittsburgh sleep quality index. Türk Psikiyatr Derg.

[23] Beck AT, Ward CH, Mendelson M, Mock J, Erbaugh J (1961). An inventory for measuring depression. Arch Gen Psychiatry.

[24] Hisli N (1988). Beck depresyon envanteri’nin geçerliği üzerine bir çalışma. Psikoloji Dergisi.

[25] Beck AT, Epstein N, Brown G, Steer RA (1988). An inventory for measuring clinical anxiety: psychometric properties. J Consult Clin Psychol.

[26] Ulusoy M, Sahin NH, Erkmen H (1998). Turkish version of the beck anxiety inventory: psychometric properties. J Cogn Psychother.

[27] Young RC, Biggs JT, Ziegler VE, Meyer DA (1978). A rating scale for mania: reliability, validity and sensitivity. Br J Psychiatry.

[28] Karadağ F, Oral T, Yalçin FA, Erten E (2001). Reliability and validity of Turkish translation of young mania rating scale. Turk Psikiyatri Derg.

[29] Kay SR, Flszbeln A, Qpjer LA (1967). The positive and negative syndrome scale (PANSS) for schizophrenia. Schizophr Bull.

[30] Kostakoğlu E, Batur S, Tiryaki A, Göğüş A (1999). Pozitif ve Negatif Sendrom Ölçeğinin (PANSS) Türkçe uyarlamasının geçerlilik ve güvenilirliği. Turk Psikol Derg.

[31] Monti JM, Torterolo P, Perumal SRP (2017). The effects of second generation antipsychotic drugs on sleep variables in healthy subjects and patients with schizophrenia. Sleep Med Rev.

[32] Benson KL (2015). Sleep in schizophrenia: pathology and treatment. Sleep Med Clin.

[33] Chan MS, Chung KF, Yung KP, Yeung WF (2017). Sleep in schizophrenia: a systematic review and meta-analysis of polysomnographic findings in case-control studies. Sleep Med Rev.

[34] Melo MCA, Garcia RF, Linhares Neto VB, Sá MB, Mesquita LMF, Araújo CFC (2016). Sleep and circadian alterations in people at risk for bipolar disorder: a systematic review. J Psychiatr Res.

[35] Cohrs S (2008). Sleep disturbances in patients with schizophrenia: impact and effect of antipsychotics. CNS Drugs.

[36] Ross CA, Margolis RL, Reading SAJ, Pletnikov M, Coyle JT (2006). Neurobiology of schizophrenia. Neuron.

[37] Maletic V, Raison C (2014). Integrated neurobiology of bipolar disorder. Front Psychiatry.

[38] Monti JM, Monti D (2004). Sleep in schizophrenia patients and the effects of antipsychotic drugs. Sleep Med Rev.

[39] Peirson SN, Foster RG, Colwell CS (2015). Circadian medicine.

[40] Maier W, Zobel A, Wagner M (2006). Schizophrenia and bipolar disorder: differences and overlaps. Curr Opin Psychiatry.

[41] Bora E (2016). Differences in cognitive impairment between schizophrenia and bipolar disorder: considering the role of heterogeneity. Psychiatry Clin Neurosci.

[42] Meyer N, Faulkner SM, McCutcheon RA, Pillinger T, Dijk DJ, MacCabe JH (2020). Sleep and circadian rhythm disturbance in remitted schizophrenia and bipolar disorder: a systematic review and meta-analysis. Schizophr Bull.

[43] Doghramji K, Jangro WC (2016). Adverse effects of psychotropic medications on sleep. Psychiatr Clin North Am.

[44] Pritchett D, Wulff K, Oliver PL, Bannerman DM, Davies KE, Harrison PJ (2012). Evaluating the links between schizophrenia and sleep and circadian rhythm disruption. J Neural Transm.

[45] Geoffroy PA, Samalin L, Llorca PM, Curis E, Bellivier F (2016). Influence of lithium on sleep and chronotypes in remitted patients with bipolar disorder. J Affect Disord.

[46] Bellivier F, Geoffroy PA, Etain B, Scott J (2015). Sleep- and circadian rhythm-associated pathways as therapeutic targets in bipolar disorder. Expert Opin Ther Targets.

[47] Bazil CW (2003). Effects of antiepileptic drugs on sleep structure: are all drugs equal?. CNS Drugs.

[48] Yetkin S, Aydın H, Özgen F, Sütcigil L, Bozkurt A (2011). Sleep architecture in schizophrenia patients. Turk Psikiyatri Derg.

[49] Boudebesse C, Geoffroy PA, Henry C, Germain A, Scott J, Lajnef M (2015). Links between sleep and body mass index in bipolar disorders: an exploratory study. Eur Psychiatry.

[50] Homel P, Casey D, Allison DB (2002). Changes in body mass index for individuals with and without schizophrenia, 1987-1996. Schizophr Res.

[51] Thomson D, Berk M, Dodd S, Rapado-Castro M, Quirk SE, Ellegaard PK (2015). Tobacco use in bipolar disorder. Clin Psychopharmacol Neurosci.

[52] Gelenberg AJ, Leon J, Evins AE, Parks JJ, Rigotti NA (2008). Smoking cessation in patients with psychiatric disorders. Prim Care Companion J Clin Psychiatry.

